# Protein profiling reveals consequences of lifestyle choices on predicted biological aging

**DOI:** 10.1038/srep17282

**Published:** 2015-12-01

**Authors:** Stefan Enroth, Sofia Bosdotter Enroth, Åsa Johansson, Ulf Gyllensten

**Affiliations:** 1 Department of Immunology, Genetics, and Pathology, Biomedical Center, SciLifeLab Uppsala, Uppsala University, SE-75108 Uppsala, Sweden; 2 Department of Medical Sciences, Uppsala University, SE-75185 Uppsala, Sweden

## Abstract

Ageing is linked to a number of changes in how the body and its organs function. On a molecular level, ageing is associated with a reduction of telomere length, changes in metabolic and gene-transcription profiles and an altered DNA-methylation pattern. Lifestyle factors such as smoking or stress can impact some of these molecular processes and thereby affect the ageing of an individual. Here we demonstrate by analysis of 77 plasma proteins in 976 individuals, that the abundance of circulating proteins accurately predicts chronological age, as well as anthropometrical measurements such as weight, height and hip circumference. The plasma protein profile can also be used to identify lifestyle factors that accelerate and decelerate ageing. We found smoking, high BMI and consumption of sugar-sweetened beverages to increase the predicted chronological age by 2–6 years, while consumption of fatty fish, drinking moderate amounts of coffee and exercising reduced the predicted age by approximately the same amount. This method can be applied to dried blood spots and may thus be useful in forensic medicine to provide basic anthropometrical measures for an individual based on a biological evidence sample.

Human ageing is associated with a number of changes in how the body and its organs function^1^. Among visible signs of ageing are greying of hair, changes in posture and loss of skin elasticity^2^,^3^. Less noticeable signs include hearing loss, increase in blood pressure or sarcopenia^4^. On the molecular level, ageing is associated with numerous processes, such as telomere length reduction, changes in metabolic and gene-transcription profiles and an altered DNA-methylation pattern^5^,^6^,^7^,^8^,^9^,^10^. In addition to chronological time, lifestyle factors such as smoking or stress can affect both the pattern of DNA-methylation^11^ and telomere length^12^ and thereby the aging of an individual. Ageing and lifestyle are the strongest known risk factors for many common non-communicable diseases, hence, lifestyle factors or molecular markers have been used as 5-year mortality predictors^13^,^14^. Additionally, specific food-items have been associated with lowered all cause mortality^15^. Various predictor models have been developed using measures of facial morphology^16^, physical fitness and physiology^12^,^17^, telomere length^18^ and methylation pattern^6^ to predict ones chronological age. Remarkably, some models are able to predict chronological age with correlation coefficients (R^2^) to actual age up to 0.75, and even above 0.90, when based on DNA-methylation status over 353 or 71 CpG-sites^6^,^19^. Comparisons of the actual chronological age with the predicted age, sometimes denoted the biological age, can be used as an indicator of health status, monitor the effect of lifestyle changes and even aid in the decision on treatment strategies for cancer patients^16^,^20^. To date, no current models have explored the potential of using the plasma protein profile for age prediction. Furthermore, while lifestyle factors such as stress have been shown to affect the rate of cellular ageing^12^, to the best of our knowledge, no studies have examined the effect of a wide range of lifestyle factors, including smoking or dietary habits, on the predicted age. We have previously characterized abundance levels of 144 circulating plasma proteins using the proximity extension assay (PEA) and have found over 40% of investigated proteins to be significantly correlated with one or more of the following factors, age, weight, length and hip circumference^10^,^21^. We therefore reasoned that the plasma protein profile might also be predictive of these traits. Here we demonstrate for the first time that the profile of circulating plasma proteins can be used to accurately predict chronological age, as well as anthropometrical measures such as height, weight and hip circumference. Moreover, we used the plasma protein-based model to identify lifestyle choices that accelerate or decelerate the predicted age. The protein analysis method used has previously been applied to dried blood spot material^22^. Interestingly, the ability to accurately predict anthropometrical characteristics from a dried blood spot sample could potentially be applicable in forensic investigations.

## Results

### Phenotype prediction from plasma protein profiles

**Figure 1:**
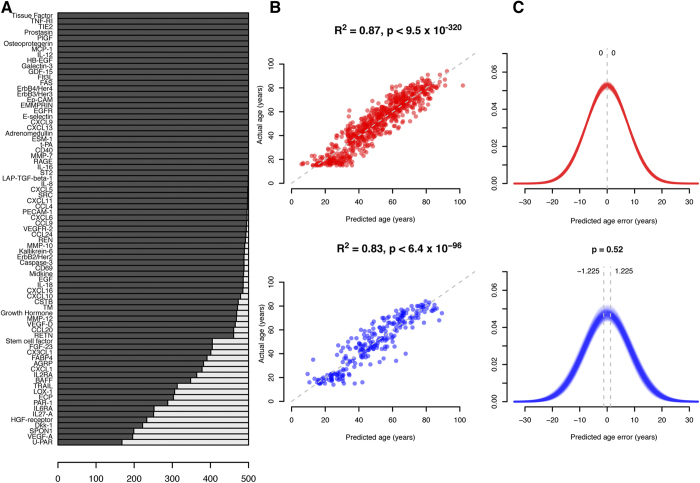
Model performance. (**A**) Inclusion-rate of proteins (number of times a protein was included in any model) in age prediction models, when executed 500 times. (**B**) Actual age (y-axis) vs. predicted age (x-axis) for one model, with training set in red and test set in blue. P-values indicate significance rate for correlation calculated using Spearman’s method. (**C**) Distribution of errors for all 500 separate execution times overlaid, with training set in red and test set in blue. Vertical dashed lines indicate the 2.5% and 97.5% quartiles of the distribution of the average error from each of the 500 separate runs, respectively. P-values for test-set represent two-sided differences of error distribution in test set vs. training set, calculated using Wilcoxon Ranked Sum test.

**Figure 2:**
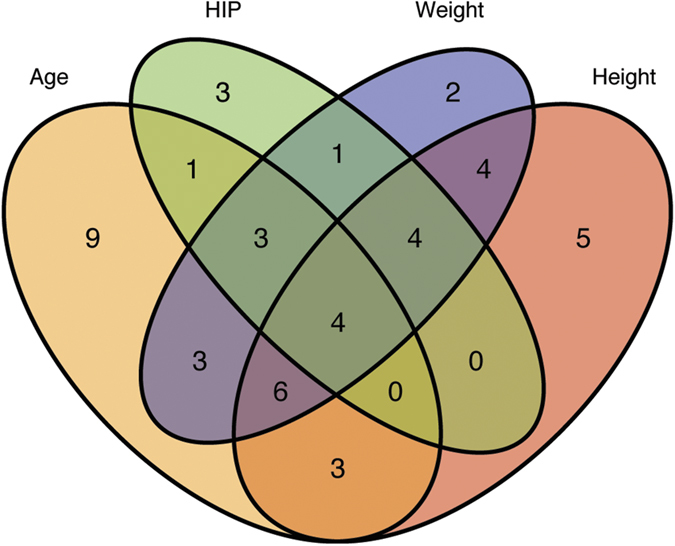
Protein overlap in core models. Overlaps between proteins present in each of the four core models predicting Age, Hip Circumference (HIP), Weight and Height.

We have previously quantified abundance levels of circulating plasma proteins from cardiovascular and cancer biomarker panels using the highly sensitive protein extension assay (PEA)^10^,^21^ in 976 individuals from the Northern Swedish Population Health Study (NSPHS). Seventy-seven of these protein measurements were used to build models to predict chronological age, weight, height and hip circumference. Prediction models were built using generalized linear models with penalized maximum likelihoods as implemented by the glmnet-package^23^ in R^24^ and models were optimized using a 10-fold cross-validation scheme on 75% of the observation and subsequently evaluated using the remaining 25% (see Methods for details). We repeated the process 500 times and recorded which proteins were selected in the model. As expected, individual variation in protein abundance values and the distribution of phenotypes, gave rise to some variation in the proteins selected to be part of the final model. On average 68 of the 77 proteins were included in the model predicting age (Fig. 1A, Table 1). In total, all 77 proteins were included at least once in any of the age predicting models and a core set of 29 proteins was present in all models. The models for age, height, weight and hip circumference performed well on the test and training sets (Table 1) and summary statistics (including protein inclusion statistics) for all models and traits are reported in Supplementary tables 2–5. The models predicted chronological age with an R^2^ = 0.83, while predicting weight (R^2^ = 0.48), height (R^2^ = 0.34) and hip circumference (R^2^ = 0.60) with somewhat lower correlation coefficients. An example of the correlation between chronological and predicted age for one model is shown in Fig. 1B, and the distribution of prediction errors for 500 age models in Fig. 1C. In the test sets, 95% of the average errors for each of the models were within +/− 1.23 years and there was no statistically significant difference (p = 0.52, Wilcoxon Ranked Sum test) between the distribution of errors in the training and test sets, indicating that the models were not over-fitted to the training data. In terms of accuracy, the plasma protein profile predicted chronological age within 5.0 years, weight within 6.8 kg, height within 4.7 cm and hip circumference within 5.1 cm, for 50% of the observations. Additional performance measurements for the models are shown in Supplementary Figures 1–3. We also evaluated the performance of the models when restricted to a core set of proteins that were included in all models for each trait (Table 1). Interestingly, the models based on the core set of proteins showed similar performance statistics as the models using the full set of proteins, suggesting that a smaller set of proteins can capture most of the phenotype variation. This observation was also confirmed by an analysis of the fraction of variance of the traits that can be explained by individual and combined proteins included in the prediction models (Supplementary Figure 4, Supplementary Tables 2–5). An analysis of the overlap between the proteins that were present in the four core-models showed that only 4 proteins (Fig. 2) were common between all models. These were Tissue plasminogen activator (tPA), Tumor necrosis factor receptor 1 (TNFR1), the Receptor tyrosine-protein kinase ErbB-3 (ErbB3) and Endothelial cell-specific molecule 1 (ESM-1). None of the genes coding for these proteins have been implicated in a recent GWAS for variation in human adult height^25^. In our material, out of the four proteins common to all models, ESM-1 explains the largest proportion of the variance seen in height (9.8%, Supplementary Table 4). ESM-1 is mainly expressed in endothelial cells in lung and kidney tissue but circulates in the bloodstream^26^. We have found no evidence relating ESM-1 to height in the literature but speculate that circulating levels of ESM-1 could be a reflection of lung volume, which is correlated to height^27^. Notably, none of the four proteins in common to the traits are among the set of proteins explaining the largest fraction of variance in the four traits (Supplementary Tables 2–5).

The proteins included in the study represent a non-random selection of the proteome since they are based on biomarker panels for cancer and cardiovascular disease. We therefore evaluated the distribution of superfamilies relative to the human proteome using the International Protein Sequence Resource (PIR) database. We found a significant overrepresentation (p < 0.05, Bonferroni adjusted) of 3 such families among the 77 proteins analysed (PIRSF002522:CXC chemokine, PIRSF001950:small inducible chemokine, C/CC type and PIRSF000619:TyrPK_EGF-R, Supplementary Table 6). In the core set of proteins used in the prediction models, only one family was shown to be overrepresented (PIRSF000619:TyrPK_EGF-R) and only in the models predicting age, weight and height. We repeated our model after removing any protein that was annotated within this family and found performance to remain unchanged (Table 1, Supplementary Figures 5–7). This suggests, however, that the non-random selection of proteins included in the analysis does not significantly contribute to the performance of the models.

### Lifestyle choices affect the biological age

**Figure 3:**
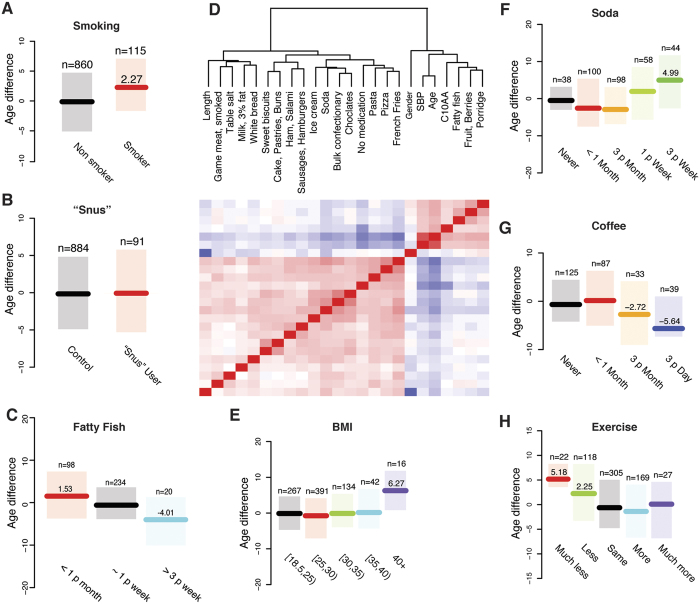
Effects of lifestyle factors on predicted age. (**A**) Smoking. Age predicting model trained on non-smokers and applied to smokers. (**B**) Snus. Snus is Swedish wet tobacco. Age predicting model trained on non-snus-users and applied to snus-users. (**C**) Fatty fish. Age prediction model trained on individuals with the most common consumption of fatty fish (Salmon, Whitefish and Herring) applied to groups with other levels of fatty fish consumption. Analysis restricted to individuals between 20 and 50 years of age. (**D**) Significant correlations between Soda consumption and other phenotypic traits in the study cohort, with red colour indicating positive and blue negative correlations. (**E**) BMI. Model trained on individuals with normal BMI (18.5–24.9) and applied to individuals with higher BMI. Analysis restricted to individuals over 20 years of age. (**F**) Soda. Age prediction model trained on individuals that do not drink soda and applied to groups with different levels of soda consumption. Analysis restricted to individuals between 20 and 50 years of age. (**G**) Coffee. Age prediction model trained on non-coffee drinkers and applied to groups with different levels of coffee consumption. Analysis restricted to individuals between 20 and 50 years of age. (**H**) Exercise. Model trained on individuals reporting that they are as active on their free-time as other individuals in their age-group, and applied to individuals that reporting to be much less, less, more or much more active than individuals in their age-group. **(A–C,E–H).** Specifically written out predicted phenotype differences imply a statistically significant (p < 0.05, two-sided Wilcoxon Ranked Sum test) change compared to the control group (coloured black). All other differences have a p > 0.05. All actual phenotype differences have a p > 0.05.

The ability to use the plasma protein profile to accurately predict age allowed us to examine the effect of lifestyle choices on the predicted phenotype (age). We first studied smoking by comparing data on 115 individuals in the study cohort who self-reported as smokers with 860 individuals that reported as non-smokers. Smoking status was used to split the cohort into training and test sets, and an age-prediction model was built using the non-smokers. This model predicted smokers to be on average 2.3 years older (Fig. 3A, p < 1.8 × 10^−4^, Wilcoxon Ranked Sum test) than their chronological age, even though the two groups do not differ in chronological age (p > 0.9, Wilcoxon Ranked Sum test). Usage of the Swedish wet tobacco product “snus” did not alter the predicted age (Fig. 3B, p > 0.5, Wilcoxon Ranked Sum test).

Body mass index (BMI) is used to classify obesity and we examined the impact of BMI on predicted age by training a model on individuals with normal weight (BMI between 18.5 and 25) and applying this to higher BMI-intervals (Fig. 3E). We observed that a BMI less than 40 does not alter the predicted versus chronological age, however individuals with a BMI over 40 were predicted to be on average 6.3 years older than their chronological age (p < 4.2 × 10^−3^, Wilcoxon Ranked Sum test).

Over 1000 phenotypic traits have been measured in our study cohort, including lifestyle factors such as dietary habits. Many of the 284 lifestyle and anthropometrical variables were, however, not independent. This is illustrated for dietary items in Fig. 3D, where variables which are significantly correlated (p < 0.05/284^2^ = 6.2 × 10^−7^, Spearman’s Rho) with soda consumption are shown. For instance, consumption of sweets (Bulk confectionery, R = 0.40, p < below machine precision (BMP)), French fries (R = 0.40, p < BMP), pizza (R = 0.25, p < 8.9 × 10^−16^) and white bread (R = 0.25, p < 3.6 × 10^−15^) were all positively correlated with soda consumption, while consumption of fatty fish (R = −0.18, p < 1.8 × 10^−7^), porridge (R = −0.19, p < 5.6 × 10^−9^) and berries (R = −0.19, p < 5.4 × 10^−9^), as well as chronological age (R = −0.38, p < 3.5 × 10^−34^), were all found to be negatively correlated with soda consumption. In light of these findings, individual dietary variables should be viewed as lifestyle indicators, and differences in plasma protein abundance is not necessarily the effect of a single food item. Nevertheless, we trained a model on non-soda drinkers and predicted the age of soda drinkers stratified by consumption. Since soda-consumption is known to be age-correlated, we included only individuals between 20 and 50 years of age, restricting the analysis to categories with at least 25 individuals. Individuals with high soda consumption were predicted to be significantly older than their chronological age (Fig. 3F, p < 9.6 × 10^−3^, Wilcoxon Ranked Sum test). There was no statistical difference in actual chronological age between individuals when stratified on soda consumption (p > 0.1, Wilcox Ranked Sum test).

In addition, we trained an age predicting model on the consumption of fatty fish. Using the most common consumption frequency (once per week) as controls, the age of individuals consuming fatty fish at least 3 times per week were predicted to be lower than their chronological age (Fig. 3C, p < 1.2 × 10^−2^, Wilcoxon Ranked Sum test), whilst individuals with little or no consumption were predicted to be older than their chronological age (Fig. 3C, p < 4.3 × 10^−2^, Wilcoxon Ranked Sum test). There was no statistical difference in chronological age between individuals when stratified on fatty fish consumption (p > 0.05). Remarkably, the same pattern was found for coffee consumption. The age-prediction model was trained on non-coffee drinkers and applied to the remaining individuals. Individuals reporting a consumption of between 3 to 6 cups of coffee per day were predicted to be on average 5.6 years younger than their chronological age (Fig. 3G, p < 4.0 × 10^−2^, Wilcoxon Ranked Sum test). This analysis was also restricted to individuals between 20 and 50 years of age, and as before there was no statistical difference in chronological age between groups based on consumption of coffee (p > 0.1, Wilcox Ranked Sum test). Finally, we studied self-reported exercise, where participants compared their own level relative to individuals of the same age in the community (peers). We trained the model using individuals that exercise at similar levels as their peers, and applied it to other exercise categories. Individuals that exercised less or much less than their peers had a significantly higher predicted age (+2.3 years, p < 1.2 × 10^−2^ versus +5.2 years, p < 7.9 × 10^−4^, Wilcox Ranked Sum test). There was no difference in actual chronological age between the different exercise groups (p > 0.6, Wilcox Ranked Sum test) (Fig. 3H). None of the individual groups in any of the lifestyles investigated showed any significant (Bonferroni adjusted p > 0.05, Breusch-Pagan test) dependency between the predicted age and the actual age.

**Figure 4:**
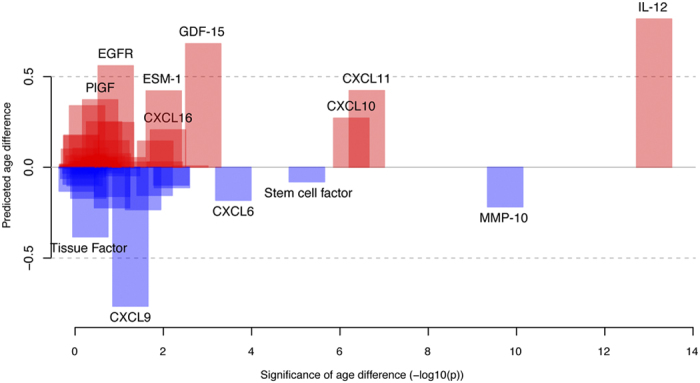
Effect of single proteins on predicted age in smokers. The age predicting model was trained on non-smokers and applied to smokers. The Y-axis shows the contribution of each protein to the total age-difference between predicted and chronological age in smokers, based on the change in protein levels between the two groups. Red (blue) colour corresponds to a positive (negative) contribution to the age in smokers compared to non-smokers. The X-axis depicts the statistical significance of that contribution for each protein (two-sided Wilcoxon Ranked Sum test, −log10(p)).

The contribution of an individual protein to the age model and the difference between groups (e.g. smokers vs. non-smokers) was in most cases modest, and an increase in protein abundance was shown to have either an additive or subtractive effect on the predicted age (Supplementary Tables 1–6). This is illustrated using the effect of individual proteins on predicted age of smokers versus non-smokers. The majority of proteins contributed a small positive or negative effect on the predicted age (Fig. 4). Some proteins however, such as the cytokines CXCL9 and CXCL10, mediated relatively large effects (on average +0.27 years in smokers compared to non-smokers, p < 5.6 × 10^−7^ and −0.77 years, p < 5.6 × 10^−2^ respectively). Both CXCL9 and CXCL10 have previously been shown to be down-regulated in response to cigarette smoke extract compared to control samples in human monocyte-derived macrophages^28^. In our age prediction model, the coefficient (β) for CXCL9 was positive while negative for CXCL10 and both abundance levels were found to be higher in non-smokers compared to smokers. Therefore, the contribution from CXCL9 to the predicted age was lower in smokers compared to non-smokers, while higher in smokers compared to non-smokers for CXCL10. Notably, IL-12 was found to contribute the largest effect (on average, +0.82 years in smokers compared to non-smokers, p < 7.6 × 10^−14^, Wilcoxon Ranked Sum test). For IL-12 the sign of the coefficient (β) in the age prediction model was negative, meaning that smokers have lower levels of IL-12, which in turn contributes to a higher predicted age compared to non-smokers.

### Limitations of the study

The NPSHS cohort used consists exclusively of western European ethnicity and therefore the models used here need not be representative of other populations. The sample size is moderate which restricts statistical evaluation of relationships between different lifestyle choices or stratifications on these. Finally, the NSPHS is a cross-sectional study and we lack follow-up data that could have been used to study relationships between longevity, mortality and the age prediction carried out here.

## Discussion

A number of molecular markers have been used to study the ageing process and to compare chronological age with the predicted age, also termed biological age. The definition of biological age has been a matter of debate for decades^29^ as have the biomarkers used to define it^30^. We have shown that the plasma protein abundance profile is highly predictive of both chronological age and basic anthropometrical traits. In examining the effect of lifestyle choices we choose to compare chronological age with the predicted age, in order to avoid using the somewhat unclear concept of biological age.

The proteins included in our study have not been selected because they belong to pathways or processes known to be involved in biological ageing, or in the development of the anthropometrical traits studied. Instead, we used protein panels designed as research tools for the discovery and validation of biomarkers for cancer and cardiovascular disease. The changes in the protein profile seen are therefore not likely to be drivers of the ageing process but merely a reflection of this process at the metabolic level. The strong correlation between traits and the abundance profile of proteins not known to be involved in their development, underscores the likelihood that other proteins exist that exhibit similar strong correlations with age. For instance, strong correlations have recently been described^31^ between chronological age and the abundance of a number of cytokines not included in our study. Additionally, analysis on proteins known to have a direct involvement in cellular ageing, e.g. telomerases and methylases, are likely to improve the models and provide even more powerful predictions.

The plasma protein profile described herein is highly accurate in predicting chronologic age, while somewhat less accurate for weight, height and hip circumference. This can be seen in the analysis of the fraction of variance of the traits that is explained by the proteins measured. In the combined model, the 77 proteins explain 85.6% of the variance in age, but only between 51.1% and 65.1% of the variance in weight, height and hip circumference. It is not known how different disease states affect the individual protein levels, and thereby the precision of the prediction models used. From a biological standpoint, some of the individual proteins might be expected to be more important for a particular trait. For instance, growth hormone followed by Fatty acid binding protein 4 (FABP4) were found to exert the strongest effect on height. For weight, the main predictive proteins were growth hormone followed by Tissue plasminogen activator (tPA). Interestingly, for age the top predictor was Osteoprotegerin, followed by Chemokine (C-X-C motif) ligand 9 (CXCL9) and growth differentiating factor (GDF-15) (Supplementary Tables 2–5). However, many of the other proteins also contributed to the different traits.

To study the consequences of lifestyle choices on the predicted age we trained a model on individuals with a specific lifestyle choice, and subsequently applied it to individuals with other choices. This enabled us to identify factors that accelerate and decelerate the predicted age by 2 to 6 years. A model trained on non-smokers predicted the age of smokers to be 2.3 years older than their actual chronological age. Whether this reflects all aspects of ageing, including risk of developing disease, or is an immediate lifespan reduction by 2.3 years cannot be determined using our cohort since we lack follow-up data, but these number are in good agreement with expected life extension following smoking cessation^32^. An interesting question is whether ceasing to smoke results in normalization of the plasma proteome profile, or if some of the changes of the protein profile are permanent? Our study is cross-sectional rather than longitudinal, and we therefore lack the possibility to examine if the effect of smoking or any other lifestyle choice is reversible or permanent. The largest effect on age seen by smoking is mediated by IL-12. Lower levels of IL-12 have previously been observed in smokers compared to non-smokers^33^,^34^, whilst the levels in moist-tobacco users and non-smokers have been found to be comparable^35^. Kroening *et al.*^33^, suggest that the suppression of IL-12 is due to oxidative stress rather than the nicotine component in cigarette smoke, which is in concordance with our observations. Previous studies have also shown a relationship between IL-12 levels and responses to smoking triggered by the immune system^36^. This suggests an acute response that could be reversible, but further studies are needed to elucidate this.

Our method allowed us to examine the consequences of lifestyle choices on the predicted age with high statistical confidence on a group level. Furthermore, the models provide the ability to predict effects at the individual level. That said, additional studies in larger cohorts are needed in order to increase the accuracy of prediction further. We envisage that having established the effect of lifestyle factors on predicted age, analyses of the plasma protein profile could be used to motivate lifestyle changes for individuals at risk of developing non-communicable disease, by monitoring the effect of such changes on the predicted age. Pointing to the effect of individual lifestyle factors is complicated by the correlation between variables. Even so, it has recently been shown that sugar-sweetened beverages causes earlier menarche in US girls^37^, demonstrating that dietary effects are not only confined to specific molecular events but can have a significant impact on the development of the body. Such dietary choices can have direct downstream consequences on the disease risk of an individual. For example, the risk of breast cancer is known to increase by 5% for each year of early onset of menarche as a consequence of longer lifetime exposure to oestrogen^38^,^39^,^40^.

The plasma protein profile may also be valuable in forensic medicine. Age and gender prediction in forensic medicine have been proposed based on DNA-methylation or N-glycan levels^41^,^42^. The PEA-technology we used for profiling of protein levels requires only 1 μl of plasma or serum for analysis of up to 92 proteins, and is applicable to dried whole blood samples^22^. Our results demonstrate that our protein profile can be used to predict a number of traits with high accuracy. In light of our findings we propose that such a method may be useful for predicting basic anthropometrical characteristics in forensic investigations. Ultimately, this possibility rests on the assumption that the protein profile is sufficiently stable over time. Future studies of the stability of individual proteins in biological samples found on crime scenes are called for. Our core-models for predicting chronological age, weight, height and hip circumference, were based on a total of 48 proteins. This suggests that only a small, specific, set of proteins might be sufficient to capture sufficient amounts of the variation in these traits to be useful for anthropometrical predictions.

## Methods

### Samples

The Northern Sweden Population Health Study (NSPHS) was initiated in 2006 to provide a health survey of the population in the parish of Karesuando, county of Norrbotten, Sweden, and to study the medical consequences of lifestyle and genetics. In the first phase, 719 individuals participated (KA06 cohort) and in a second phase, another 350 individuals from a neighboring village (Soppero) were recruited in 2009 (KA09 cohort). Here, 974 individuals with were included out of which 510 are female and 464 male with ages ranging from 14 to 94, mean (+/− standard deviation) height was 164 (9.6) cm, weight 72.3 (15.3) kgs and hip circumference 97 (13.5) cm. For each participant, blood samples were drawn (serum and plasma) and stored at −70 °C on site. A questionnaire was used to collect data on medications and lifestyle. The anthropometrical measurements were carried out, and the questionnaire was filled in, at the local health care center in the presence of the local district nurse.

### Ethical considerations

The NSPHS study was approved by the local ethics committee at the University of Uppsala (Regionala Etikprövningsnämnden, Uppsala, 2005:325) in compliance with the Declaration of Helsinki^43^. All participants gave their written informed consent to the study including the examination of environmental and genetic causes of disease. In cases where the participant was not of age, a legal guardian signed additionally. The procedure that was used to obtain informed consent and the respective informed consent form has recently been discussed in light of present ethical guidelines^44^.

### Plasma protein profiles

The Protein Extension Assay (PEA) has previously been used to measure the plasma protein levels of 144 proteins in the NSPHS cohort. The protein abundance levels were measured using the Olink Proseek Multiplex Oncology I^96 × 96^ and CVD I^96 × 96^ kits as previously described^10^,^21^. All assay characteristics including detection limits and measurements of assay performance and validations are available from the manufacturers webpage (http://www.olink.com/products/proseek-multiplex/downloads/data-packages).

### Model generation

Phenotype prediction models were built in R^24^ utilizing the glmnet-package^23^. The glmnet-package implements elastic net regularization, which performs variable selection and fits Intercept and Beta-values for each selected variable (protein) yielding a linear model explaining the response variable (phenotype). The glmnet-package does not allow for missing values and therefore the data set was pruned to the 77 proteins with no missing values nor measurements below detection limit in any of the individuals (Supplementary Table 1) The Gaussian model-family was used throughout and the elastic-net penalty (α) was set to 0.6.

### Model evaluation

Model performance was evaluated by randomly splitting the observations into a training set (75%) and a test set (25%). The model was optimized using a 10-fold cross validation schema over the training set using the ‘cv.glmnet’-function from the glmnet-package. A final model was built from the training set using the ‘glmnet’-function with the lambda-values returned by ‘cv.glmnet’. This model was then used to predict the response variable in the test set. The random-split into training and test set and the variable selection of the elastic net regularization can result in different proteins being selected and thus different models. The process was thus repeated 500 times and the proteins included in the models as well as model prediction errors on the training and test set were recorded. The correlation between actual and predicted values was reported for one model only. This was carried out separately for each investigated phenotype.

### Effects of lifestyle-choices

The observations were split into train and test set(s) according to e.g. smoking status or consumption patterns of specific food items. A single model was built and optimized using e.g. non-smokers and then applied to smokers and the difference between actual response variable (e.g. age) and predicted was recorded.

### Statistics and figure generation

The Protein Information Resource (PIR)^45^ superfamily overrepresentation analysis was carried out using the DAVID online resource^46^,^47^ using the whole human genome as background. Associations were considered significant if the reported Bonferroni adjusted p-values were below 0.05. All other statistical analyses were carried out in R. Tests for heteroskedasticity were carried out using the ‘bptest’ function from the ‘lmtest’^48^ R-package implementing the Breusch-Pagan test. The fraction of variance explained by a variable in a trait was calculated by fitting a linear model using the trait as response and one or several proteins as variables. Differences in phenotype distribution were examined using two-sided Wilcoxon rank tests and correlation coefficients and statistics between variables were calculated employing two-sided Spearman statistics. Heatmaps and dendrograms for clustering of correlations were calculated using the R-function ‘hclust’ with Euclidean distances and employing the Wards agglomeration method. Venn diagrams were drawn using the VennDiagram R-package^49^.

## Additional Information

**How to cite this article**: Enroth, S. *et al.* Protein profiling reveals consequences of lifestyle choices on predicted biological aging. *Sci. Rep.* **5**, 17282; doi: 10.1038/srep17282 (2015).

## Supplementary Material

Supplementary Information

Supplementary Table 1

## Figures and Tables

**Table 1:** Model performances for the traits.

Trait	Model size	Actual-predicted correlation (R^2^)			Error (mean +/− sd)		Error^e^	
		Train	Test	P-val^d^	Train	Test	Train	Test
Age	69^a^	0.87	0.83	6.4 × 10^−96^	0.0 +/− 7.6 yrs	−0.13 +/− 8.4 yrs	5.2 yrs	5.0 yrs
	29^b^	0.84	0.85	7.9 × 10^−103^	0.0 +/− 8.1 yrs	−0.66 +/− 8.3 yrs	5.2 yrs	5.4 yrs
	64^c^	0.85	0.82	4.8 × 10^−90^	0.0 +/− 8.1 yrs	0.0 +/− 8.7 yrs	5.4 yrs	5.5 yrs
Weight	60^a^	0.55	0.48	1.3 × 10^−35^	0.0 +/− 10.3 kg	0.94 +/− 10.5 kg	6.1 kg	6.8 kg
	27^b^	0.52	0.44	3.2 × 10^−32^	0.0 +/− 10.8 kg	0.61 +/− 10.6 kg	6.2 kg	7.3 kg
	56^c^	0.52	0.46	1.3 × 10^−33^	0.0 +/− 10.9 kg	−0.15 +/− 10.5 kg	6.3 kg	8.0 kg
Height	66^a^	0.52	0.34	6.2 × 10^−24^	0.0 +/− 6.8 cm	0.60 +/− 7.5 cm	4.6 cm	4.7 cm
	26^b^	0.43	0.44	3.3 × 10^−32^	0.0 +/− 7.2 cm	0.26 +/− 7.3 cm	4.6 cm	5.4 cm
	61^c^	0.46	0.35	6.0 × 10^−24^	0.0 +/− 6.9 cm	−0.28 +/− 8.0 cm	4.8 cm	5.0 cm
HIP	51^a^	0.61	0.60	2.2 × 10^−49^	0.0 +/− 8.0 cm	−0.30 +/− 8.7 cm	4.8 cm	5.1 cm
	16^b^	0.57	0.54	1.3 × 10^−42^	0.0 +/− 8.6 cm	0.80 +/− 8.7 cm	5.1 cm	5.3 cm

^a^Median number of proteins used in the models in 500 train-test splits.

^b^Core set of proteins used in all 500 train-test splits.

^c^As ^a^but excluding proteins with over-represented PIR superfamilies compared to the complete set of human proteins as background.

^d^Significance of correlation between actual and predictive values in test set.

^e^As defined in Horvath^6^, i.e. 50% of subjects classified within this error.
