# Supplementary material for: Protein profiling reveals consequences of lifestyle choices on predicted biological aging
Source: Sci Rep. 2015 Dec 1;5:17282. doi: 10.1038/srep17282 (PMC4664859; doi:10.1038/srep17282)
Supplement: Supplementary Information [file srep17282-s1.pdf]

Protein profiling reveals consequences of lifestyle choices on predicted biological aging

Stefan Enroth[1], Sofia Bosdotter Enroth[2], Åsa Johansson[1] and Ulf Gyllensten[1]

[1] Department of Immunology, Genetics, and Pathology, Biomedical Center, SciLifeLab Uppsala, Uppsala University, SE-75108 Uppsala, Sweden.

[2] Department of Medical Sciences, Uppsala University, SE-75185 Uppsala, Sweden

Supplementary Information

Supplementary Figures 2
Supplementary Tables 9

Supplementary Figures

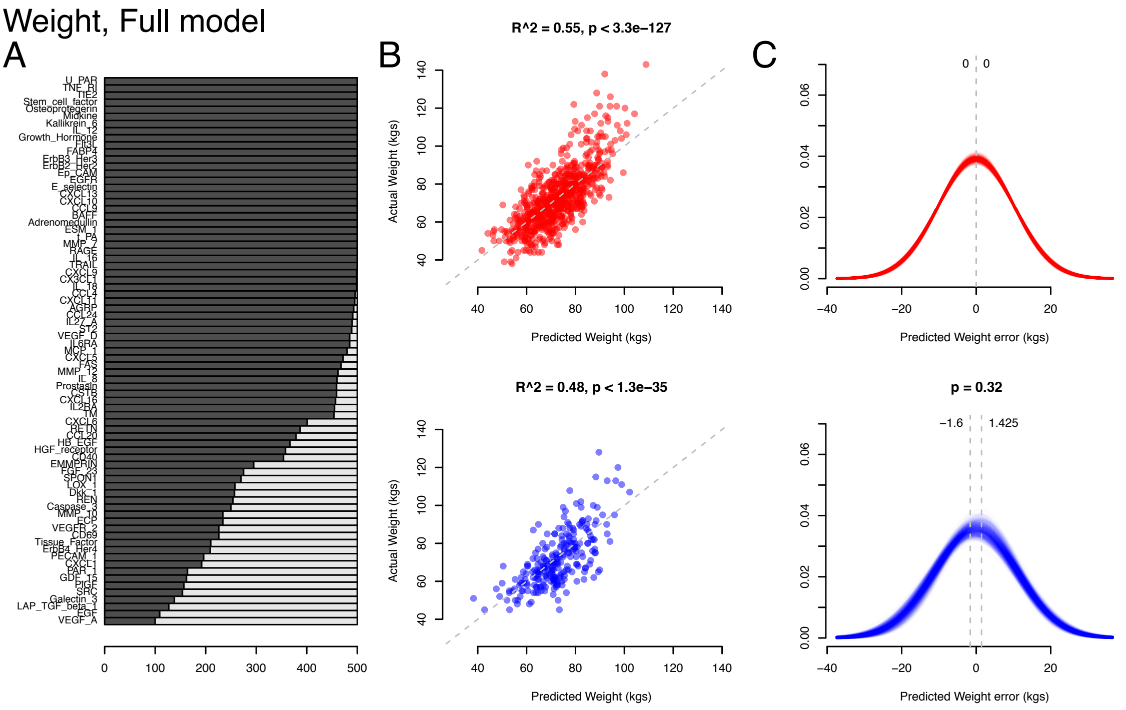

Supplementary Figure 1. Model performance. (**A**) Inclusion-rate of proteins into the weight prediction model, executed 500 times. (**B**) Actual (y-axis) vs. predicted weight (x-axis) for one model, with training set in red and test set in blue. P-values indicate significance rate for correlation calculated using Spearman's method. (**C**) Distribution of individual errors for all 500 execution times, with training set in red and test set in blue. Vertical dashed lines indicate the 2.5% and 97.5% quartiles of mean-error, respectively. P-value for test-set represent two-sided difference of error distribution in test set vs. training set, calculated using Wilcoxon Ranked Sum test.

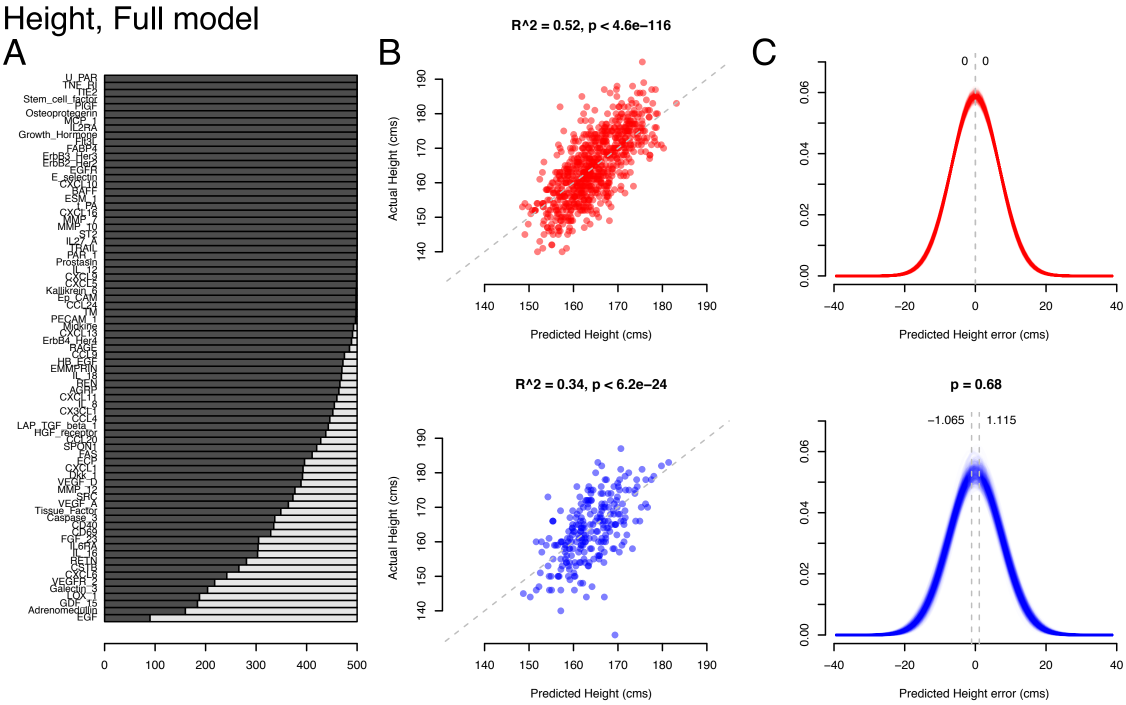

Supplementary Figure 2. Model performance. (**A**) Inclusion-rate of proteins into the height prediction model, executed 500 times. (**B**) Actual (y-axis) vs. predicted height (x-axis) for one model, with training set in red and test set in blue. P-values indicate significance rate for correlation calculated using Spearman's method. (**C**) Distribution of individual errors for all 500 execution times, with training set in red and test set in blue. Vertical dashed lines indicate the 2.5% and 97.5% quartiles of mean-error, respectively. P-value for test-set represent two-sided difference of error distribution in test set vs. training set, calculated using Wilcoxon Ranked Sum test.

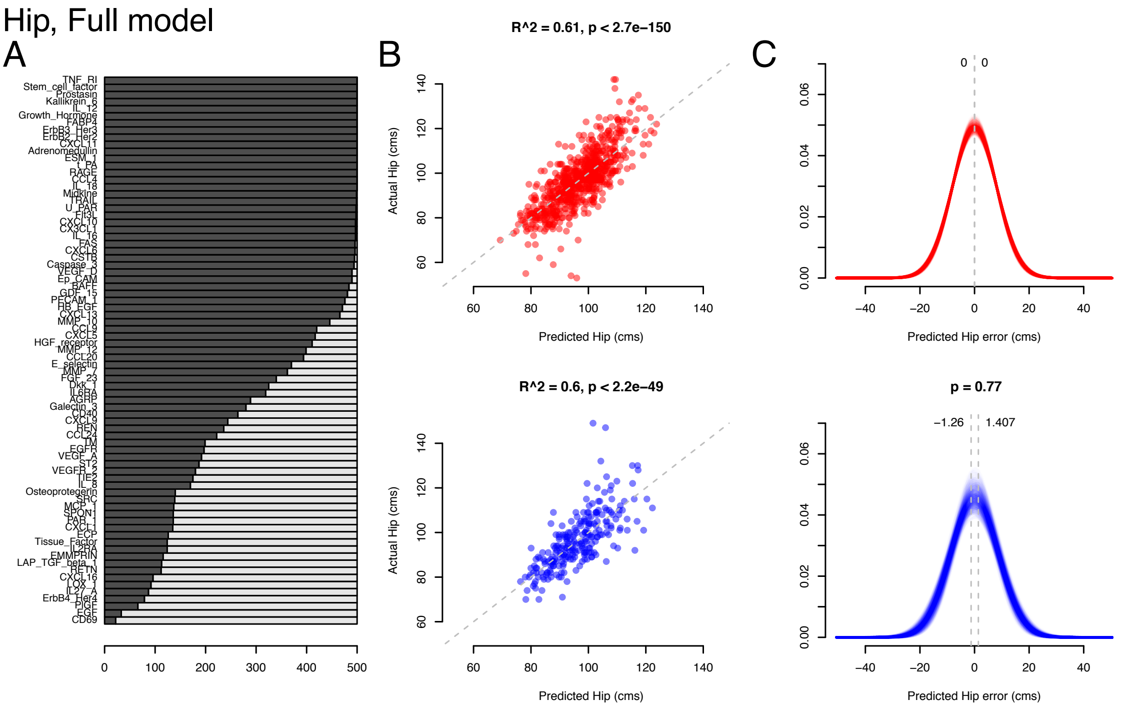

Supplementary Figure 3. Model performance. (**A**) Inclusion-rate of proteins into the hip-circumference prediction model, executed 500 times. (**B**) Actual (y-axis) vs. predicted hip-circumference (x-axis) for one model, with training set in red and test set in blue. P-values indicate significance rate for correlation calculated using Spearman's method. (**C**) Distribution of individual errors for all 500 execution times, with training set in red and test set in blue. Vertical dashed lines indicate the 2.5% and 97.5% quartiles of mean-error, respectively. P-value for test-set represent two-sided difference of error distribution in test set vs. training set, calculated using Wilcoxon Ranked Sum test.

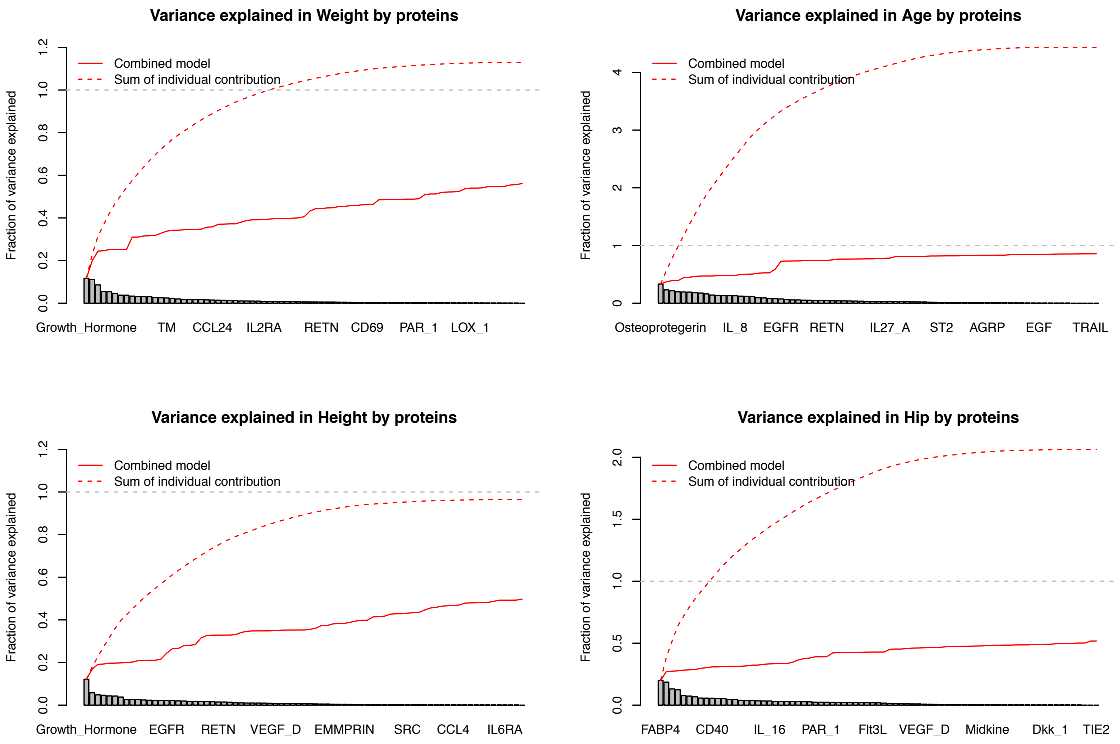

Supplementary Figure 4. Analysis of variance explained. Each panel depicts the individual contribution to variance explained (grey bars, Supplementary Tables 2-5) in the analyzed phenotype by the proteins included in the prediction models. The solid red curve denotes the combined variance explained by taking multiple proteins into account simultaneously. The dashed red curve shows a hypothetical cumulative sum of the individual proteins' fraction of variance explained. The horizontal grey dashed line indicates 100% of variance seen in the phenotypes.

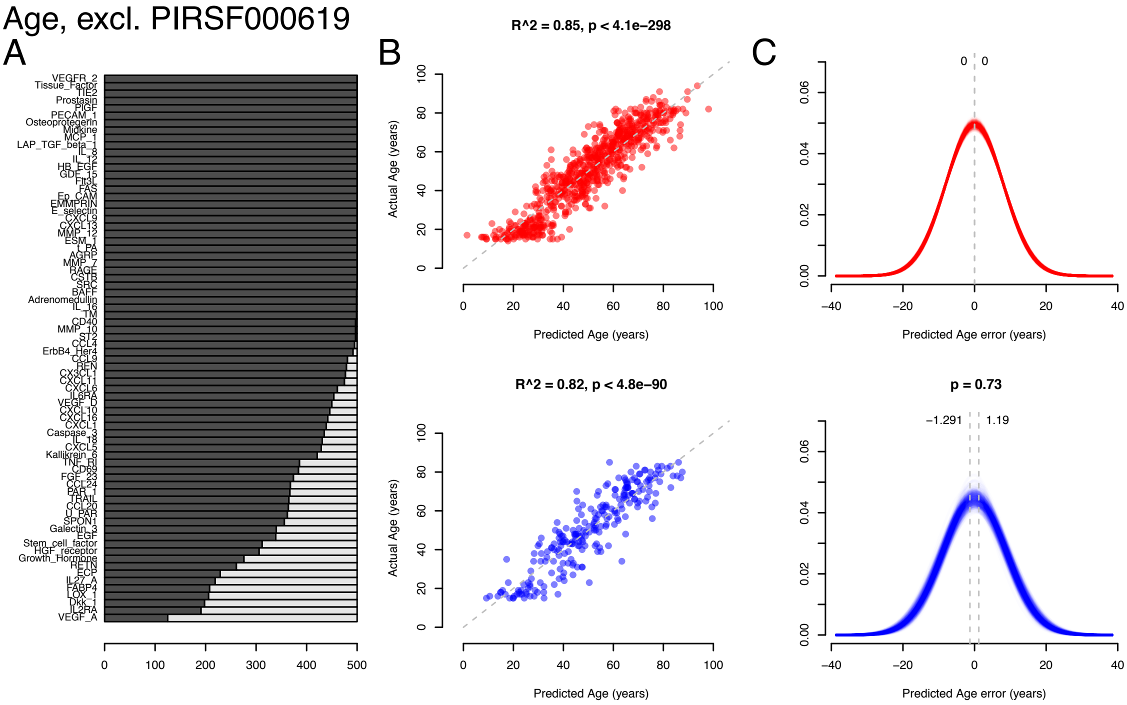

Supplementary Figure 5. Model performance. (**A**) Inclusion-rate of proteins into the age prediction model, executed 500 times when excluding proteins annotated to the PIR Superfamily PIRSF00619 (TyrPK_EGF-R). (**B**) Actual (y-axis) vs. predicted age (x-axis) for one model, with training set in red and test set in blue. P-values indicate significance rate for correlation calculated using Spearman's method. (**C**) Distribution of individual errors for all 500 execution times, with training set in red and test set in blue. Vertical dashed lines indicate the 2.5% and 97.5% quartiles of mean-error, respectively. P-value for test-set represent two-sided difference of error distribution in test set vs. training set, calculated using Wilcoxon Ranked Sum test.

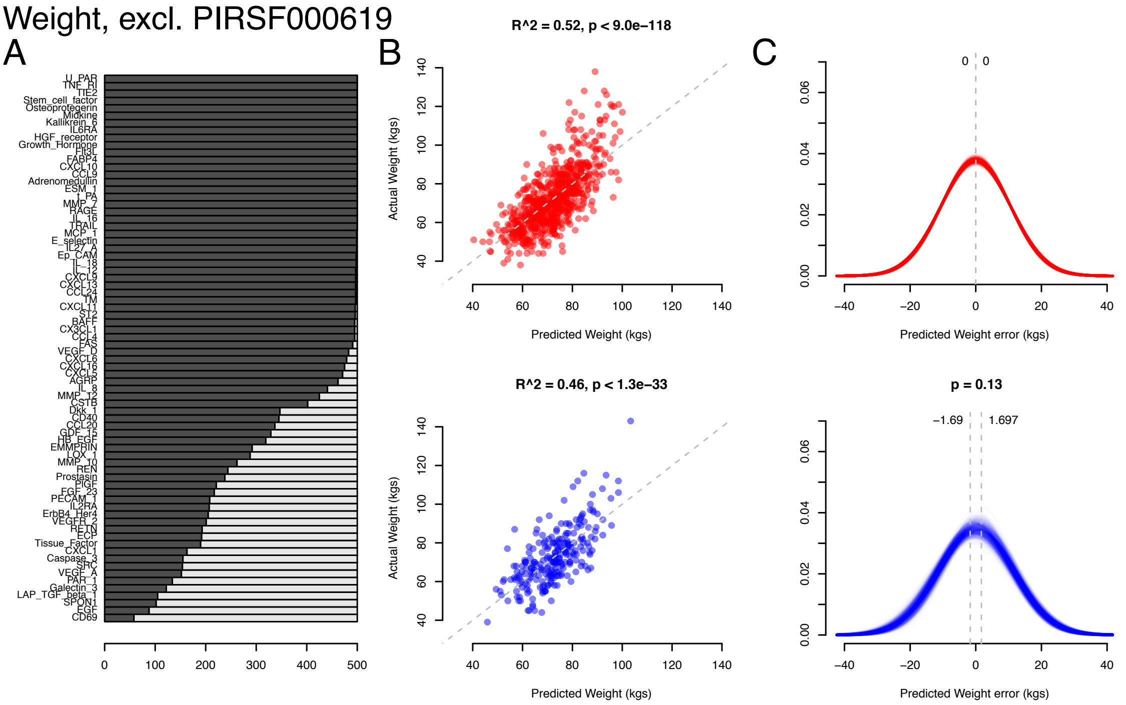

Supplementary Figure 6. Model performance. (**A**) Inclusion-rate of proteins into the weight prediction model, executed 500 times when excluding proteins annotated to the PIR Superfamily PIRSF00619 (TyrPK_EGF-R). (**B**) Actual (y-axis) vs. predicted weight (x-axis) for one model, with training set in red and test set in blue. P-values indicate significance rate for correlation calculated using Spearman's method. (**C**) Distribution of individual errors for all 500 execution times, with training set in red and test set in blue. Vertical dashed lines indicate the 2.5% and 97.5% quartiles of mean-error, respectively. P-value for test-set represent two-sided difference of error distribution in test set vs. training set, calculated using Wilcoxon Ranked Sum test.

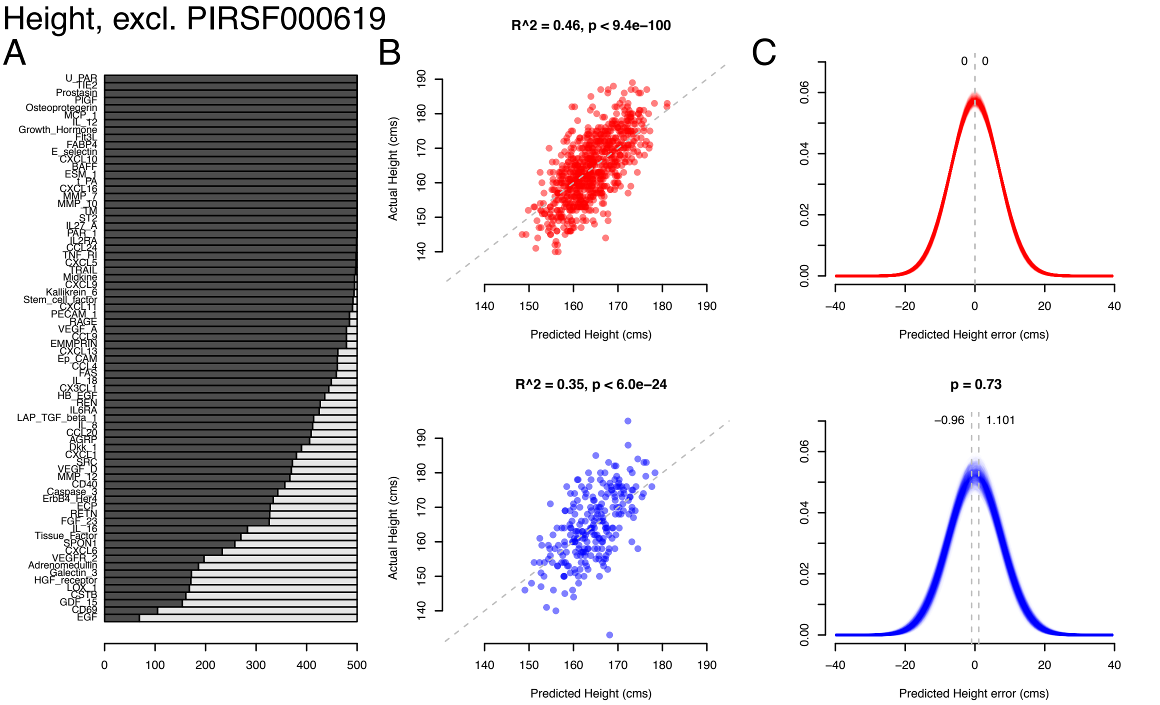

Supplementary Figure 7. Model performance. (**A**) Inclusion-rate of proteins into the height prediction model, executed 500 times when excluding proteins annotated to the PIR Superfamily PIRSF00619 (TyrPK_EGF-R). (**B**) Actual (y-axis) vs. predicted height (x-axis) for one model, with training set in red and test set in blue. P-values indicate significance rate for correlation calculated using Spearman's method. (**C**) Distribution of individual errors for all 500 execution times, with training set in red and test set in blue. Vertical dashed lines indicate the 2.5% and 97.5% quartiles of mean-error, respectively. P-value for test-set represent two-sided difference of error distribution in test set vs. training set, calculated using Wilcoxon Ranked Sum test.

Supplementary Tables

Protein	% Included	1st Quartile (B)	mean (B)	3rd Quartile (B)	Variance Explained in Phenotype
Osteoprotegerin	100	8,766236243	9,172751216	9,601602014	0,332085005
CXCL9	100	3,401820797	3,697165855	3,99454215	0,231014334
GDF_15	100	3,751470104	4,002514649	4,226930464	0,214734385
Adrenomedullin	100	1,570243102	1,910233876	2,293791255	0,196818871
t_PA	100	3,340448773	3,600949313	3,856858117	0,181381115
Flt3L	100	2,268558947	2,63154529	2,960208668	0,175913777
CD40	100	1,860490103	2,293792235	2,717731044	0,140972285
MMP_7	100	1,448447108	1,687815715	1,935987296	0,136350571
FAS	100	2,312478285	2,526096253	2,725306385	0,134007875
MCP_1	100	2,552497487	2,961711641	3,323745424	0,119415979
Tissue_Factor	100	13,16947012	13,72580193	14,22429627	0,118878355
TNF_RI	100	-5,128733991	-4,417120498	-3,689156581	0,077760755
EGFR	100	-12,47486864	-11,92967136	-11,3564594	0,072926688
IL_16	100	1,348506242	1,550915768	1,772750718	0,051205155
ErbB4_Her4	100	5,991975088	6,497240406	6,975421424	0,040909779
HB_EGF	100	2,007030942	2,76155874	3,451909812	0,028997561
Prostasin	100	-4,228951028	-3,855411625	-3,436234223	0,027593725
EMMPRIN	100	-14,88624952	-14,14270969	-13,41762081	0,027058458
Galectin_3	100	2,685897745	3,159585	3,62293536	0,022249094
RAGE	100	-5,227098497	-4,84411493	-4,47816776	0,016766218
ST2	100	-1,669292518	-1,419599866	-1,169036037	0,01380534
TIE2	100	-3,74187974	-3,130258256	-2,550020844	0,013302912
E_selectin	100	-2,283814053	-2,065168879	-1,833516962	0,009605031
PlGF	100	-4,604236329	-4,175635736	-3,753773868	0,008600103
ErbB3_Her3	100	-6,578370625	-5,756195049	-4,994476171	0,007802262
Ep_CAM	100	-2,420901571	-2,262787286	-2,099744694	0,00381101
ESM_1	100	-3,632590981	-3,25520271	-2,901592708	0,000232988
CXCL13	100	-1,501612106	-1,334319268	-1,181055954	0,00019545
IL_12	100	-1,573766226	-1,372005057	-1,154046029	4,41E-06

IL_8	99,8	0,641258992	0,812136711	0,991605934	0,129335765
LAP_TGF_beta_1	99,8	-1,596237187	-1,349436754	-0,822643499	0,027511431
CXCL5	99,6	0,621596848	0,796434369	0,978412349	0,002874675
SRC	99,6	-1,563125853	-1,251443454	-0,971704882	0,000135173
CCL4	99,4	0,641676255	0,821497988	1,005355409	0,063342155
CXCL11	99,4	-1,354720584	-1,103424049	-0,870636805	0,041316993
CXCL6	99,2	0,616837154	0,819742845	1,034152677	0,00630865
PECAM_1	99,2	1,460257824	1,896198784	2,377235603	9,31E-05
CCL19	99	-0,522406075	-0,397443135	-0,260182676	0,003368858
VEGFR_2	98,8	-3,084786695	-2,375562965	-1,601865125	0,05214445
CCL24	98,6	0,322934522	0,4634696	0,598178792	0,010496105
REN	98,4	-0,673281525	-0,515585276	-0,34149217	0,056632595
MMP_10	98	-0,770116154	-0,585771229	-0,407884063	0,040131133
Kallikrein_6	97,8	0,711594176	1,098533755	1,45065752	0,025193622
CD69	97,6	1,372651629	1,770121081	2,181027158	0,006542293
Caspase_3	97,6	-1,491665355	-1,173956783	-0,864440578	0,000646106
ErbB2_Her2	97,6	1,633379193	2,221584661	2,801830116	0,000102905
Midkine	97,4	-1,589987688	-1,205824626	-0,836106269	0,032117628
IL_18	97,2	0,446043163	0,678571902	0,895626369	0,047875352
EGF	97,2	-1,360822499	-1,044506869	-0,709075077	0,000881204
CXCL16	97	1,124183234	1,679593912	2,220499001	0,022897494
CXCL10	95,8	-0,495440479	-0,368670307	-0,234140062	0,091585452
CSTB	94,6	0,688523294	1,02392027	1,35600289	0,194358451
TM	94,2	-1,887684297	-1,388482116	-0,869577441	0,021519091
Growth_Hormone	93,8	-0,177316595	-0,128937363	-0,076283079	0,009426954
MMP_12	93,6	0,270807584	0,481624434	0,66540718	0,192505284
VEGF_D	93	-0,8315996	-0,601214736	-0,361910591	0,001672764
CCL20	92,2	-0,473049774	-0,355238342	-0,221785973	0,080188909
RETN	92,2	-1,089387248	-0,791008102	-0,469026482	0,045855033
FGF_23	81	-0,39267656	-0,270699609	-0,127304827	0,036656636
Stem_cell_factor	81	0,26045757	0,519911717	0,764253494	0,015343965

CX3CL1	80,2	0,361552321	0,689970702	0,935869903	0,034650917
FABP4	78,2	0,13961056	0,388494744	0,600315823	0,161199764
AGRP	76,4	-0,544943043	-0,369857898	-0,142941227	0,006619744
CXCL1	75,6	-0,575848379	-0,374512639	-0,159214501	0,012673538
IL2RA	72,8	-0,973026429	-0,685059601	-0,299291985	0,026488604
BAFF	69,6	-0,887471429	-0,632015239	-0,321326176	0,000173855
TRAIL	62,6	-0,720571206	-0,305578613	0,004769406	1,95E-05
LOX_1	61,2	0,105779876	0,274203817	0,427721865	0,048778597
ECP	60,6	-0,031194218	0,050874571	0,157668902	0,038327132
PAR_1	57,6	0,057060051	0,384844189	0,727997551	0,056931191
IL6RA	50,4	-0,132575272	0,076198647	0,302421702	0,00085424
IL27_A	50,2	-0,519554545	-0,088829198	0,281751871	0,027281226
HGF_receptor	46,6	-0,789093903	-0,102569574	0,683054328	0,005972864
Dkk_1	44,4	-0,127337608	0,121253038	0,366474722	0,001285116
SPON1	39,8	-0,526053898	-0,138262652	0,306877845	0,132933675
VEGF_A	39,2	0,014457306	0,330264324	0,594539796	0,093911391
U_PAR	33,6	-0,251714147	0,113798117	0,455332909	0,121627292

Supplementary Table 2. Summary of model parameters for the 500 instances run of predicting age. Column "% Included" reports percentages of models where a specific protein was included. Columns "1st Quartile (B)", "mean (B)" and "3rd Quartile (B)" reports summary statistics on intervals of the coefficients (Beta) for each protein. Column "Variation Explained in Phenotype" reports the fraction of variance [0,1] explained by each protein.

Protein	% Included	1st Quartile (B)	mean (B)	3rd Quartile (B)	Variance Explained in Phenotype
Growth_Hormone	100	-0,960686226	-0,877883918	-0,795853524	0,116769266
t_PA	100	3,690837044	4,016768889	4,340693012	0,110843105
FABP4	100	2,349651396	2,655771447	2,966748721	0,085760232
Adrenomedullin	100	5,458994709	5,992921343	6,544403304	0,054684646
ErbB2_Her2	100	7,146608306	7,965795702	8,775827501	0,037655946
ESM_1	100	-6,840777246	-6,359208597	-5,870759023	0,033099298
TNF_RI	100	9,480423981	10,4004185	11,24513377	0,032007459
RAGE	100	-4,053823641	-3,582533312	-3,122536787	0,02537174
IL_16	100	1,245477143	1,55132428	1,891184147	0,022721761
CCL19	100	1,018205741	1,201624829	1,370559093	0,018087776
CXCL10	100	1,087754812	1,226827716	1,369092508	0,017659175
TRAIL	100	2,378647989	2,943775433	3,533558869	0,017380975
Stem_cell_factor	100	-4,389382783	-3,96014213	-3,521584069	0,014061478
E_selectin	100	-1,922741341	-1,594883834	-1,263508862	0,010412332
EGFR	100	4,880693164	5,681976137	6,394892423	0,009998964
TIE2	100	3,240518434	3,92114832	4,589029031	0,007967207
Midkine	100	-3,212843399	-2,788793574	-2,345634742	0,005881465
ErbB3_Her3	100	-11,47460572	-10,41209977	-9,355325757	0,005844987
Ep_CAM	100	-0,996970167	-0,849207012	-0,686766166	0,0053393
CXCL13	100	-1,538722621	-1,289811588	-1,035532378	0,004804364
IL_12	100	1,401234906	1,657555185	1,912763876	0,004208863
BAFF	100	-4,413888164	-3,851892134	-3,273835419	0,002646599
Osteoprotegerin	100	-5,242111402	-4,781861258	-4,316754972	0,001652755
Flt3L	100	-3,781803699	-3,400966817	-3,018033782	0,001336835
Kallikrein_6	100	-5,253745018	-4,83213304	-4,384051114	0,000783086
U_PAR	100	-6,940287451	-6,110144523	-5,281907126	0,000469879
MMP_7	100	-2,09738959	-1,814045417	-1,505037415	1,29E-05
IL_18	99,8	1,088626091	1,414018255	1,759005655	0,053838408

CX3CL1	99,8	-2,276838367	-1,790028078	-1,301556969	0,001493912
CXCL9	99,8	-1,674776405	-1,350552781	-1,020286864	0,000121066
CCL4	99,2	0,661836238	0,914965238	1,169302164	0,027291945
CXCL11	99	-1,375853339	-1,035286797	-0,722741469	0,000102916
AGRP	98,6	-1,471655481	-1,123176502	-0,753810966	0,006393056
CCL24	98,2	0,417881765	0,623166645	0,820741502	0,014792728
IL27_A	98	-2,026441599	-1,564910477	-1,089497151	0,010252616
ST2	97,8	0,61524279	0,929081332	1,247798427	0,007052697
VEGF_D	97	-1,234054882	-0,919343773	-0,584000366	0,01552508
IL6RA	97	0,636041979	1,030324472	1,415642435	0,009795495
MCP_1	96	1,017870753	1,547653755	2,043740837	0,03756878
CXCL5	94,4	-0,764165945	-0,574238547	-0,365716505	0,000923177
FAS	93,6	0,304089918	0,573931536	0,802897862	0,019727243
MMP_12	92,4	-0,897732501	-0,658750025	-0,377752983	0,000224922
IL_8	92	0,435445397	0,713535567	0,967380302	0,008392869
CSTB	91,8	0,759615931	1,290923259	1,75946131	0,046017126
Prostasin	91,8	-1,571191241	-1,178677198	-0,721880142	0,03053929
CXCL16	91,4	-1,943004924	-1,478743177	-0,880542856	0,007114013
IL2RA	91	0,84334121	1,405992771	1,878823721	0,008529074
TM	90,8	0,883699628	1,564142564	2,18983715	0,024478576
CXCL6	80,2	0,249029664	0,512399086	0,738531459	0,001961991
RETN	77,4	0,27934497	0,612534989	0,887583445	0,005488374
CCL20	75,8	0,14237251	0,309636069	0,463242861	0,002432155
HB_EGF	73,4	0,482690052	1,046693857	1,455475284	0,003698461
HGF_receptor	71,6	-2,514836627	-1,721904394	-0,67325837	0,000420978
CD40	70,8	0,315168449	0,785521803	1,149969992	0,017730291
EMMPRIN	59	-1,730594373	-1,180035458	-0,435526605	0,000746097
FGF_23	55	-0,011408268	0,111223124	0,273248455	0,000384217
SPON1	54	0,405170149	1,108570399	1,688905958	0,000582046
LOX_1	51,6	-0,483784457	-0,319485339	-0,100093135	0,000759129
Dkk_1	51,4	-0,74899652	-0,479286253	-0,187152979	0,00453875

REN	50,8	-0,199325062	-0,026098467	0,144055407	0,013243615
Caspase_3	50	-0,433701187	-0,33319629	-0,127843378	0,002110671
ECP	46,8	-0,279465267	-0,131935253	-0,020378082	0,002095953
MMP_10	46,8	-0,107366424	0,043492373	0,188995843	0,001992565
VEGFR_2	45,2	-1,284442275	-0,78972565	-0,266995149	0,013612012
CD69	45,2	-0,607199196	-0,437217362	-0,142211773	0,003419895
Tissue_Factor	42	0,317212477	0,731797967	1,034717239	0,006154069
ErbB4_Her4	41,8	-1,016311856	-0,68981904	-0,230665896	0,000995973
PECAM_1	39,2	0,140054259	0,570661186	0,923418642	0,007615288
CXCL1	38,4	-0,215036875	0,034906959	0,283572598	0,001364911
PAR_1	32,8	-0,434457916	-0,145638129	0,152280609	0,001762622
GDF_15	32,4	0,074790643	0,323696622	0,517903401	0,005352086
PlGF	31,4	-0,785473961	-0,537004793	-0,189707787	0,012809107
SRC	30,8	-0,079598328	0,162012552	0,361614062	0,003741923
Galectin_3	27,6	-0,132550411	0,238757732	0,698466155	0,003323977
LAP_TGF_beta_1	25,4	-0,304870251	-0,134659871	0,059509805	0,002307413
EGF	21,8	0,054446566	0,499215299	0,883632585	0,00125033
VEGF_A	20	-0,447415143	-0,006723849	0,401282268	0,03041892

Supplementary Table 3. Summary of model parameters for the 500 instances run of predicting weight. Column “% Included” reports percentages of models where a specific protein was included. Columns “1st Quartile (B)”, “mean (B)” and ”3rd Quartile (B)” reports summary statistics on intervals of the coefficients (Beta) for each protein. Column “Variation Explained in Phenotype” reports the fraction of variance [0,1] explained by each protein.

Protein	% Included	1st Quartile (B)	mean (B)	3rd Quartile (B)	Variance Explained in Phenotype
Growth_Hormone	100	-0,743321662	-0,685244001	-0,630701592	0,121511006
FABP4	100	-2,47392805	-2,298561836	-2,117148609	0,057941703
MMP_7	100	-3,006492225	-2,801362058	-2,575410777	0,048180128
Osteoprotegerin	100	-3,623129846	-3,248599946	-2,885277479	0,046281933
U_PAR	100	-3,516397481	-2,971812854	-2,384511086	0,037945278
IL27_A	100	-2,062789778	-1,719812107	-1,370782632	0,026854058
Flt3L	100	-3,053817075	-2,750904408	-2,452454218	0,022981769
EGFR	100	3,695218009	4,214294726	4,752785877	0,021175994
ST2	100	1,661172576	1,859950928	2,067774025	0,021139186
BAFF	100	-3,290519412	-2,906286541	-2,548490507	0,019331242
TIE2	100	4,299729209	4,820303777	5,300833009	0,017297516
ErbB2_Her2	100	2,245145206	2,77367425	3,322659011	0,010544306
ESM_1	100	-2,224949721	-1,966550031	-1,694293116	0,009842276
MMP_10	100	0,693681163	0,863333937	1,041668554	0,005654314
CXCL10	100	0,752895541	0,858948092	0,960637994	0,005184013
PAR_1	100	1,600962578	2,002439022	2,402713899	0,003902488
TRAIL	100	1,201773763	1,564625771	1,914350228	0,002210232
CXCL16	100	-3,979355254	-3,559947032	-3,129296332	0,001975882
PlGF	100	1,694794792	2,008384332	2,309052164	0,00169708
E_selectin	100	-2,168179024	-1,952987791	-1,755755248	0,001091226
IL2RA	100	2,39321401	2,785073987	3,236504131	0,000981553
ErbB3_Her3	100	-5,385289901	-4,671639028	-3,941252082	0,000644094
TNF_RI	100	3,527890428	4,318149967	5,070306661	0,000536006
Stem_cell_factor	100	-1,606649069	-1,338743902	-1,08553007	0,000223062
MCP_1	100	1,995479406	2,31913544	2,691360418	0,000201479
t_PA	100	1,350915833	1,590483944	1,805611829	7,61E-06
CXCL9	99,8	-0,979003099	-0,77466849	-0,561252693	0,042737915
CXCL5	99,8	-0,851296436	-0,688585773	-0,510705113	0,015973702

IL_12	99,8	-1,022993298	-0,827758857	-0,638258244	0,007060893
Prostasin	99,8	1,093783729	1,463219319	1,799033982	0,004264861
CCL24	99,6	0,36901288	0,499415901	0,625034508	0,009912808
Kallikrein_6	99,6	-1,646209577	-1,345004297	-1,043247874	0,004134083
TM	99,6	1,156009373	1,563282585	1,953725829	0,003208581
Ep_CAM	99,6	-0,566306884	-0,437126868	-0,30474702	0,000579021
PECAM_1	99,4	0,964485274	1,313907941	1,670531282	0,001659698
Midkine	98,6	0,84686266	1,18505071	1,507782355	0,01082769
CXCL13	98,2	-0,657293021	-0,51293652	-0,364669898	0,01449604
ErbB4_Her4	97,8	-2,267559776	-1,766517397	-1,323834509	0,001525806
RAGE	97	0,562883215	0,906499238	1,220715699	0,000308704
CCL19	95	0,259934031	0,371156085	0,475918855	0,001704834
HB_EGF	94,4	0,704313499	1,30419318	1,690905416	0,009795526
EMMPRIN	94	-2,968893296	-2,25075024	-1,480702383	0,004009997
IL_18	93,8	-0,704012531	-0,520207292	-0,324242443	0,000494156
REN	93,2	-0,572032392	-0,42506301	-0,268302048	0,001922051
AGRP	92,8	-0,829590051	-0,606899007	-0,349416335	0,000918313
CXCL11	92	-0,64875437	-0,487244655	-0,294397547	0,007649937
IL_8	91	0,214758309	0,385282255	0,542368196	0,007947554
CX3CL1	90,4	-1,068214727	-0,78624301	-0,450284256	0,008849949
CCL4	89,2	0,204401343	0,380062339	0,545493744	0,000602597
LAP_TGF_beta_1	88,6	-1,639483578	-0,951633651	-0,287405835	0,006846096
HGF_receptor	87,6	1,39504437	2,418264216	3,390329437	0,009557628
CCL20	85,6	-0,327554509	-0,225228834	-0,118188694	0,024304889
SPON1	84	0,545296026	1,176235194	1,635841575	0,026631902
FAS	82,2	0,17752461	0,369249308	0,538000647	0,000388219
ECP	79,2	-0,291952454	-0,212893596	-0,10135214	0,013700744
CXCL1	78,6	-0,52978761	-0,354633921	-0,159944998	0,021691398
Dkk_1	78,4	-0,783521145	-0,555248492	-0,266682096	0,003184882
VEGF_D	77,8	-0,436966297	-0,299277734	-0,126534353	0,008456834
MMP_12	75,4	-0,407195128	-0,267844044	-0,111321309	0,043202556

SRC	74,6	-0,608483802	-0,415479544	-0,18805812	0,001554794
VEGF_A	72,8	-1,433879435	-1,010044093	-0,478965648	0,005173937
Tissue_Factor	69,8	0,37769903	0,764797093	1,098642503	0,000425476
Caspase_3	67,4	0,161269519	0,422383978	0,624227191	0,001531215
CD40	67	0,256167779	0,63198666	0,92096983	0,006392863
CD69	65,8	-0,83846391	-0,563186613	-0,239350403	0,000630554
FGF_23	61	-0,208857703	-0,102048046	0,001988859	0,022081208
IL6RA	60,8	0,084231999	0,277442344	0,461100193	0,0001955
IL_16	60,6	0,035749803	0,188382816	0,331545715	8,96E-05
RETN	56,2	-0,35385318	-0,1943497	-0,028625689	0,014668069
CSTB	53,2	0,25752517	0,517259489	0,740229163	0,017166923
CXCL6	48,4	-0,08057603	0,059540802	0,198836655	0,001619999
VEGFR_2	43,6	0,018987906	0,429448988	0,787602656	0,017896262
Galectin_3	40,8	0,00309625	0,201689413	0,478510556	0,006914131
LOX_1	37,6	-0,131144195	0,07558885	0,311056683	0,019825639
GDF_15	36,8	0,063797869	0,219392323	0,374693848	0,026848463
Adrenomedullin	32	-0,206507882	0,026041675	0,257886605	0,017877348
EGF	18	0,000362021	0,212613922	0,42537354	4,42E-05

Supplementary Table 4. Summary of model parameters for the 500 instances run of predicting height. Column "% Included" reports percentages of models where a specific protein was included. Columns "1st Quartile (B)", "mean (B)" and "3rd Quartile (B)" reports summary statistics on intervals of the coefficients (Beta) for each protein. Column "Variation Explained in Phenotype" reports the fraction of variance [0,1] explained by each protein.

Protein	% Included	1st Quartile (B)	mean (B)	3rd Quartile (B)	Variance Explained in Phenotype
FABP4	100	3,889421991	4,088479331	4,30810858	0,200185438
t_PA	100	3,239762833	3,47889962	3,75050942	0,185636336
Adrenomedullin	100	4,301418554	4,660151618	4,996878249	0,130731388
IL_18	100	1,182403451	1,474353861	1,72892327	0,076654446
CCL4	100	0,959987238	1,164002709	1,371103582	0,066999645
TNF_RI	100	3,965415928	4,623059621	5,286894266	0,056323325
Prostasin	100	-2,714870942	-2,363854082	-2,020781874	0,03368609
Growth_Hormone	100	-0,489835192	-0,43005945	-0,371497955	0,027342595
Stem_cell_factor	100	-2,180206562	-1,823749293	-1,463930057	0,023627481
RAGE	100	-4,38186898	-4,064921127	-3,725495767	0,022867135
ErbB2_Her2	100	5,078362752	5,734858086	6,267856697	0,019308956
ESM_1	100	-5,739822124	-5,430267387	-5,111975661	0,014656484
IL_12	100	1,21553461	1,414805835	1,604287031	0,01099714
CXCL11	100	-1,304899732	-1,102523751	-0,909519355	0,005753134
Kallikrein_6	100	-2,389042889	-2,051637045	-1,704430008	0,000933065
ErbB3_Her3	100	-10,64349369	-9,839139839	-8,997804128	3,24E-06
TRAIL	99,8	1,355012795	1,743844476	2,132440144	0,016339444
Midkine	99,8	-2,187590917	-1,841250787	-1,509301571	0,002940983
U_PAR	99,6	-2,796314179	-2,269933529	-1,696084961	0,028267461
Flt3L	99,6	-1,488904208	-1,194065439	-0,876009435	0,019157049
CXCL10	99,4	0,344839088	0,436969831	0,542096082	0,044524065
IL_16	99,4	0,705779078	0,958984034	1,216584362	0,03346299
CX3CL1	99,4	-1,789416626	-1,43763777	-1,084425818	0,000759783
FAS	99,2	0,477863949	0,679294071	0,858873693	0,052126051
CXCL6	99,2	0,699145433	0,95967809	1,217763959	0,007257597
CSTB	99	0,963346267	1,373226177	1,738124214	0,123930291
Caspase_3	98,8	-0,619027887	-0,483160863	-0,289289423	0,000409007

Ep_CAM	98	-0,476262937	-0,358934587	-0,245195093	0,009972631
VEGF_D	98	-0,901819807	-0,684099434	-0,462516605	0,00782098
BAFF	96,8	-1,383567494	-1,034194394	-0,619333685	0,000757983
GDF_15	96,2	0,259274076	0,436839824	0,588725576	0,07318701
PECAM_1	95,2	-1,170012716	-0,877568734	-0,519114823	0,003399751
HB_EGF	94,2	0,76735376	1,291401384	1,762928953	0,004869957
CXCL13	93,2	-0,57715987	-0,43629133	-0,267433845	0,000882364
MMP_10	89,2	-0,532074415	-0,376322102	-0,191581036	0,002299968
CCL19	84	0,154600666	0,31781136	0,445636224	0,019662411
CXCL5	83,4	-0,442842471	-0,315994163	-0,16714235	8,10E-06
HGF_receptor	82,2	-2,203451304	-1,584856098	-0,868896423	0,004450102
MMP_12	79,8	-0,533457735	-0,372540428	-0,162393742	0,034358494
CCL20	78,8	0,132318683	0,269320241	0,385405933	0,029561963
E_selectin	74	-0,51554177	-0,365696908	-0,197923733	0,013813343
MMP_7	72,4	-0,395194509	-0,281127204	-0,129642339	0,029046603
FGF_23	68	0,116293722	0,246545419	0,360187201	0,023236709
Dkk_1	65	0,164804339	0,411081171	0,569712543	0,000774965
IL6RA	63,8	0,197717159	0,443167872	0,626707959	0,004512912
AGRP	57,8	-0,693111305	-0,447372646	-0,12960587	0,006212953
Galectin_3	56	0,32141619	0,73114863	1,019064412	0,020473444
CD40	52,8	0,232897371	0,590820196	0,852926936	0,055594841
CXCL9	48,8	-0,484679051	-0,339243048	-0,13344854	0,037088693
REN	47,2	0,026659741	0,134798616	0,251504492	0,044894145
CCL24	44,4	-0,185306015	-0,094196329	-0,007832428	0,001287451
TM	39,8	0,150822317	0,541830483	0,776667864	0,038542906
EGFR	39,4	0,2282003	0,592495127	0,946528076	0,00059049
VEGF_A	38,4	0,249695756	0,680800474	1,029262327	0,05442751
ST2	37,4	-0,185058088	-0,006092799	0,15589874	0,006638277
VEGFR_2	36	-0,968188272	-0,650553712	-0,258600506	0,000549552
TIE2	35	0,215712447	0,625270112	0,956793209	5,15E-07
IL_8	34	-0,160053027	-0,018299984	0,133035135	0,02949107

Osteoprotegerin	28	-0,557306849	-0,376101349	-0,126885519	0,057014458
SRC	27,8	0,125358691	0,3530087	0,510931775	0,002782476
MCP_1	27,4	0,026856024	0,179892728	0,412563395	0,039050862
PAR_1	27,2	0,002029797	0,181490028	0,33786326	0,02347643
SPON1	27,2	0,132134663	0,487531048	0,769936627	0,020505143
CXCL1	27	-0,0924048	0,09248901	0,318271637	0,001585325
ECP	25,2	-0,10172925	-0,011262255	0,07867821	0,018884219
Tissue_Factor	24,8	-0,842391795	-0,528760115	-0,146673118	0,009865312
IL2RA	24,8	-0,545542953	-0,378435687	-0,118879223	0,005394355
EMMPRIN	23,2	-0,947225206	-0,59232124	-0,184746668	0,002939637
LAP_TGF_beta_1	22,6	0,028553765	0,196935355	0,339526706	0,011223902
RETN	22,4	-0,362058611	-0,199451654	-0,015788055	0,029940336
CXCL16	19,2	-0,507189862	-0,320887979	-0,015371167	0,021155532
LOX_1	18,4	0,00840743	0,138832821	0,253779574	0,021870815
IL27_A	17,4	-0,00657523	0,216415745	0,46519748	0,004873676
ErbB4_Her4	15,8	0,108011743	0,469149785	0,890381429	0,000782353
PlGF	13,2	-0,739419529	-0,465439527	-0,089684808	0,026605025
EGF	6,6	0,009026955	0,241102611	0,496894572	0,000938866
CD69	4,4	-0,064245406	0,057890979	0,121445299	0,001636231

Supplementary Table 5. Summary of model parameters for the 500 instances run of predicting hip circumference. Column "% Included" reports percentages of models where a specific protein was included. Columns "1st Quartile (B)", "mean (B)" and "3rd Quartile (B)" reports summary statistics on intervals of the coefficients (Beta) for each protein. Column "Variation Explained in Phenotype" reports the fraction of variance [0,1] explained by each protein.

Analysis group	PIR Superfamily	P-value (Bonferroni adjusted)
All 77 proteins	PIRSF002522:CXC chemokine	2.4e-08
	PIRSF000619:TyrPK_EGF-R	9.7e-05
	PIRSF001950:small inducible chemokine, C/CC types	0.0014
Proteins in Age Core Model	PIRSF000619:TyrPK_EGF-R	0.0015
Proteins in Weight Core Model	PIRSF000619:TyrPK_EGF-R	0.0015
Proteins in Height Core Model	PIRSF000619:TyrPK_EGF-R	0.0012

Supplementary Table 6. Results from the analysis of overrepresented PIR Superfamilies using the DAVID online resource.
